# Associations between adherence to a Mediterranean diet and assisted reproductive techniques outcomes: a systematic review

**DOI:** 10.1093/eurpub/ckaf100

**Published:** 2025-07-11

**Authors:** Laura Martín-Manchado, Miriam Sánchez-Sansegundo, Antonio M Moya-Yeste, José A Hurtado-Sánchez, José Tuells, Ana Zaragoza-Martí

**Affiliations:** Department of Nursing, University of Alicante, Alicante, Spain; Department of Health Psychology, University of Alicante, Alicante, Spain; Gynecology and Obstetrics Service, Hospital IMED de Levante, Benidorm, Spain; Department of Nursing, University of Alicante, Alicante, Spain; Department of Community Nursing, Preventive Medicine, Public Health and History of Science, University of Alicante, Alicante, Spain; Alicante Institute for Health and Biomedical Research (ISABIAL-FISABIO Foundation), Alicante, Spain; Department of Nursing, University of Alicante, Alicante, Spain; Alicante Institute for Health and Biomedical Research (ISABIAL-FISABIO Foundation), Alicante, Spain

## Abstract

Globally, one out of six people of childbearing age experience infertility. Given the high demand for assisted reproductive techniques (ARTs), this systematic review evaluated associations between adherence to the Mediterranean diet (MD) and ART outcomes, as dietary habits can play a significant role. A systematic review was conducted by searching five databases (PubMed, ScienceDirect, Embase, Cochrane Library, and Web of Science) for studies published from 1 January 2010, to 1 January 2024, in English or Spanish. Inclusion criteria targeted patients aged 18–55 and examined intermediate and/or clinical ART outcomes. Systematic reviews, meta-analyses, and animal studies were excluded. Of the eight included articles, four found statistically significant associations between adherence to MD and positive ART outcomes, including increased number of embryos, and higher clinical and biochemical pregnancy and live birth rates. Other studies showed conflicting results. There is a potential association between adherence to the MD and ART outcomes. However, the evidence is limited, emphasizing the need for further investigation.

## Introduction

Infertility, defined as the inability of a couple to achieve pregnancy after a year of regular unprotected sexual intercourse, has become a public health issue, affecting approximately one out of six people of childbearing age worldwide [1]. Socioeconomic and cultural changes in recent years have led to a growing trend towards delayed parenthood, with couples opting to postpone childbearing until their 30s or 40s [2]. According to European Union fertility indicators, women who gave birth to their first child in 2022 had an average age of 29.7 years [3]. This trend is linked to declining oocyte quality and higher rates of comorbidities that worsen reproductive outcomes [4].

Assisted reproductive techniques (ARTs), such as *in vitro* fertilization (IVF), have become crucial in addressing infertility, accounting for approximately 1 in 20 births in developed countries [5, 6]. Despite the growing use of ART, success remains limited, with around 50% of couples failing to conceive after multiple attempts [7]. These challenges highlight the importance of identifying factors associated with ART outcomes.

Regarding the factors that can affect the outcome of ART, increasing attention is being paid to the influence of lifestyles [8]. Some studies show that an unhealthy lifestyle negatively affects oocyte and embryo quality, fertilization rate, and pregnancy rate [8]. Specifically, some modifiable factors linked to ART outcomes include lack of physical activity, exposure to endocrine disruptors such as those from cigarette smoke, chronic stress, drug and alcohol consumption, excess body weight, and dietary habits [8, 9]. In this regard, research on the influence of dietary and nutritional factors has gained popularity in recent years. However, interest has migrated from the study of isolated nutrients or food groups such as dairy or whole grains to research on dietary patterns [10].

Among the dietary patterns that have attracted considerable interest is the Mediterranean diet (MD), globally renowned for its cardiovascular and metabolic health benefits [11, 12]. Originating from countries in the Mediterranean basin, this diet is characterized by a high and varied intake of vegetables, fruits, and minimally processed whole grains, along with fish, cold-pressed olive oil, and legumes. Additionally, it involves a moderate intake of dairy products (especially cheese and yogurt) and white meats, as well as a limited amount of red and processed meats. This combination of foods not only provides a rich source of antioxidant vitamins (β-carotene, vitamin C, vitamin E), folic acid, phytochemicals, and minerals such as selenium but is also associated with a low intake of trans and saturated fatty acids [13].

Multiple epidemiological studies have found associations between the MD pattern and various positive health effects, including preventive effects against cardiovascular diseases and, more recently, neurodegenerative diseases such as Alzheimer’s [11, 14, 15]. Similarly, recent studies suggest that adherence to the MD may improve reproductive health by enhancing sperm motility and reducing female infertility-related disorders like polycystic ovary syndrome and endometriosis [16, 17]. Regarding ART outcomes, a 2010 study found that the MD increased the likelihood of pregnancy after IVF [18]. Similarly, a cohort study reported that women under 35 with high MD adherence had 2.7 times greater chances of clinical pregnancy and live birth [19]. A systematic review by Kellow *et al.* also linked the MD and other dietary patterns to improved ART outcomes but highlighted inconsistencies requiring further Research [20].

Therefore, based on the hypothesis that high adherence to the MD would be linked to better outcomes in ART (fertilization rate, implantation rate, clinical pregnancy, or live birth), the objective of this systematic review is to evaluate the associations between adherence to MD and ART outcomes. Accordingly, the research question guiding this review is: Is adherence to the MD associated with better outcomes in ART?

## Methods

A systematic literature review was conducted and reported following the PRISMA 2020 guidelines [21]. The protocol for systematic review was registered in the PROSPERO database (CRD42023471284).

### Eligibility criteria

Studies were included if they: (i) provided full-text and abstract; (ii) were in English or Spanish; (iii) published between 1 January 2010 and 1 January 2024; (iv) involved patients aged 18–55 years; (v) examined ART intermediate outcomes (oocytes retrieved, fertilization rate, embryo quality) or clinical endpoints (implantation, pregnancy, live births); and (vi) used validated tools to assess MD adherence.

Exclusion applied to: (i) unrelated topics or protocols without results; (ii) case reports, reviews, meta-analyses; (iii) conference abstracts/proceedings; (iv) studies on ART techniques other than IVF, intracytoplasmic sperm injection (ICSI), or intrauterine insemination (IUI); and (v) animal studies.

### Search strategy

**Figure 1.**
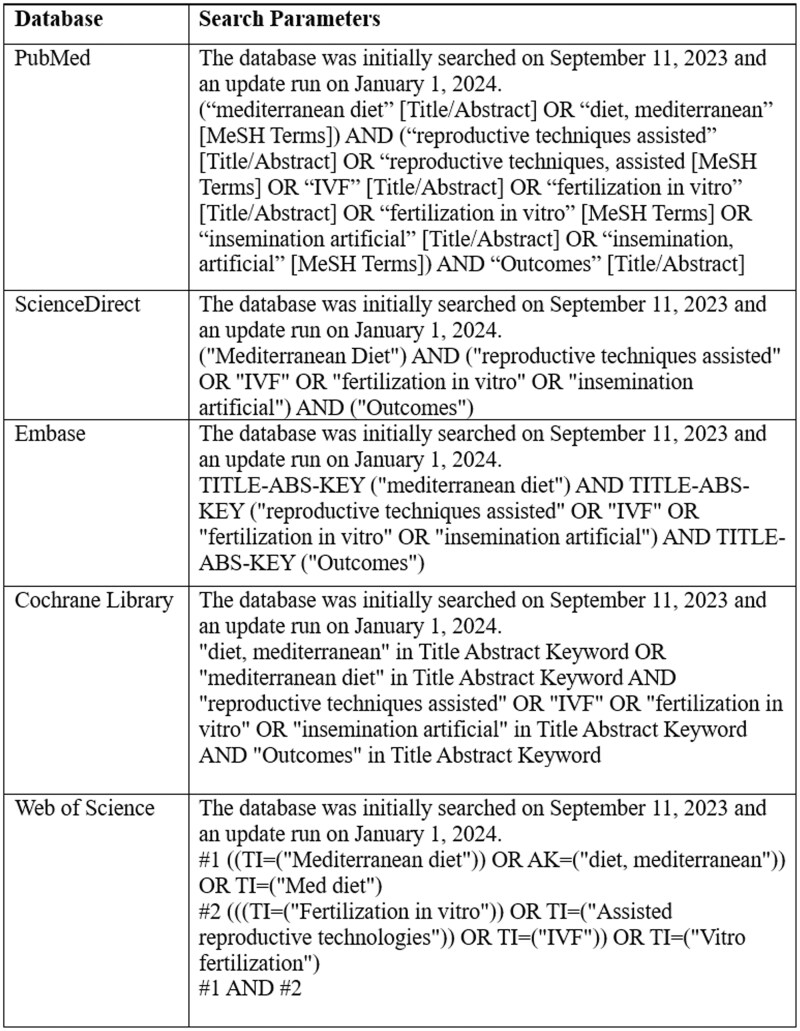
Database search strategies for a systematic review on associations between Mediterranean diet and outcomes of assisted reproductive techniques.

The search strategy aimed to identify studies published and available in full text. A comprehensive search strategy was implemented using both Medical Subject Headings (MeSH) and title/abstract terms. The following keywords were transformed into MeSH terms: ‘diet, mediterranean’, ‘reproductive techniques, assisted’, ‘fertilization *in vitro*’, and ‘insemination, artificial’, combined with the Boolean operators AND and OR. Figure 1 shows the search strategy used in each database.

### Article selection and data extraction process

The selection process involved two steps: titles and abstracts from MEDLINE (PubMed), ScienceDirect, Embase, Cochrane Library, and WOS were independently screened by two authors (L.M.M. and A.Z.M.), with disagreements resolved by a third (A.M.M.Y.). Full-text articles meeting inclusion criteria were then independently assessed by the same authors, consulting the third author as needed. Data extraction, performed by the lead author (L.M.M.), included details such as authorship, publication year, study design, sample size, participants’ age range, country, measured variables (dietary and clinical), results, and conclusions.

### Risk of bias assessment

The AXIS tool [22] was used to assess the risk of bias in cross-sectional studies. This instrument includes 20 items covering a wide range of methodological aspects, such as the clarity of study aims, appropriateness of study design, sampling strategy, sample size justification, measurement of variables, statistical methods, consistency of results, discussion of limitations, ethical approval, and conflict of interest disclosure.

For cohort studies, the Newcastle-Ottawa scale (NOS) [23] was employed. This scale evaluates eight items grouped into three key domains: selection of study groups, comparability of cohorts, and assessment of outcomes and follow-up.

Two authors (L.M.M. and A.Z.M.) independently assessed the included articles. Discrepancies were resolved by a third author (A.M.M.Y.). Inter-rater reliability was strong, with a Cohen’s kappa coefficient of 0.8.

To evaluate the overall certainty of the evidence, the Grading of Recommendations, Assessment, Development, and Evaluation (GRADE) approach [24] was applied. This method considers five domains: risk of bias, inconsistency, indirectness, imprecision, and potential for publication bias. Based on these criteria, the certainty of evidence for each outcome was classified as high, moderate, low, or very low. Any discrepancies in the assessment were resolved through discussion and consensus among the three authors (L.M.M., A.Z.M., and A.M.M.Y.).

## Results

### Selection of studies

**Figure 2.**
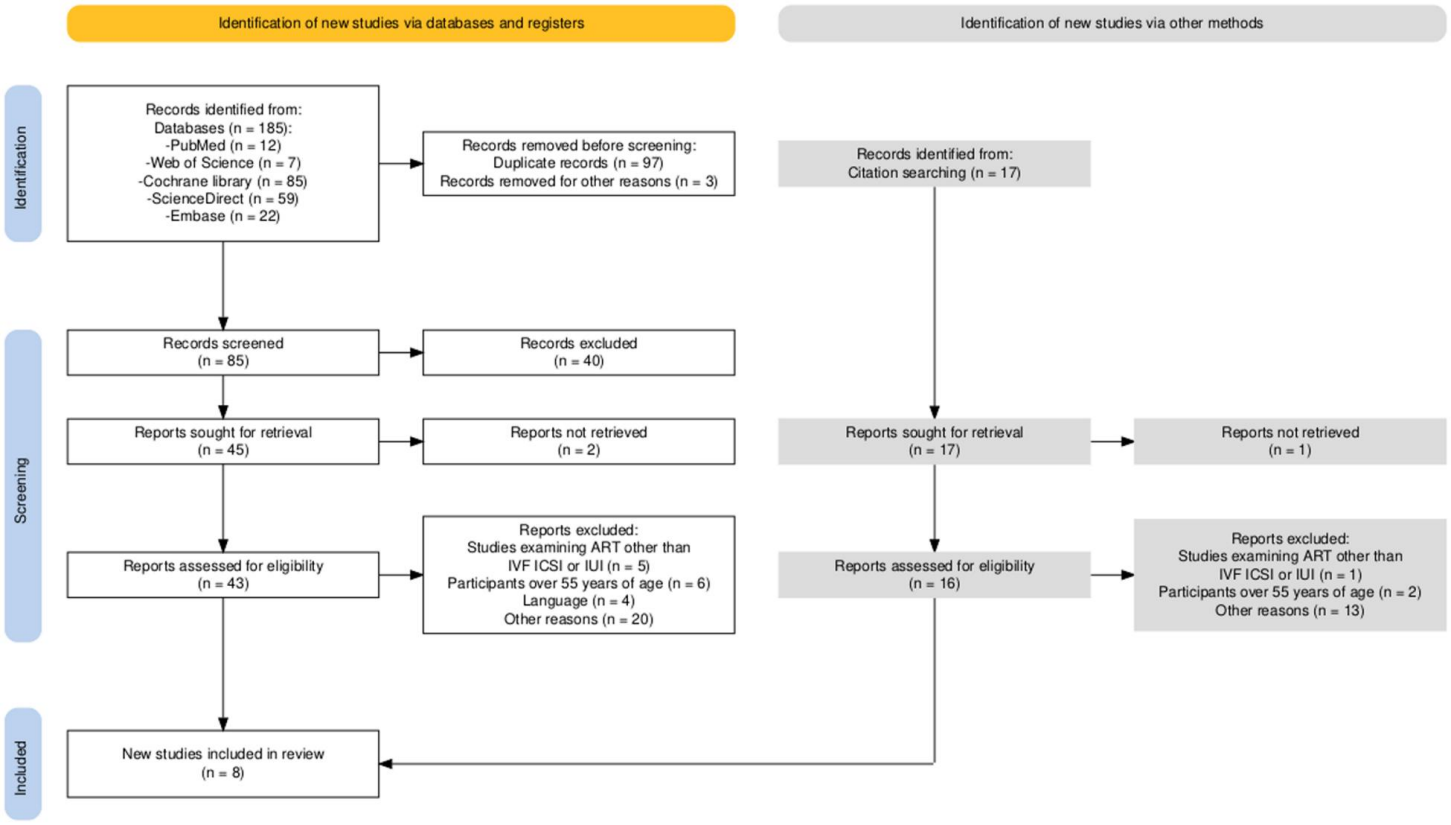
Flowchart of the literature search and results filtering for a systematic review of the associations between adherence to the Mediterranean diet and outcomes of assisted reproductive technologies.

Figure 2 summarizes the PRISMA 2020 flow diagram [21]. A total of 202 records were identified, of which 100 were excluded (97 duplicates, 3 for other reasons), leaving 102 for screening. After screening, 40 records were excluded, and 45 full-text articles were assessed for eligibility. Of these, 2 could not be retrieved, and 35 were excluded based on criteria, resulting in 8 articles included in the review.

### Characteristics of the studies

Table 1 summarizes the characteristics of the included articles. Most studies were conducted in the USA (37.5%), followed by Italy (25%), Greece, China, and the Netherlands (12.5% each). Participants ranged from 18 to 46 years, with a total of 2979 subjects (2734 women and 245 men). Seven studies (87.5%) focused on women, one (12.5%) on men, and none analysed both sexes. The majority were cohort studies (87.5%), with one cross-sectional study (12.5%).

**Table 1.** Design, characteristics, results, and conclusions of the sample of studies included in the systematic review of the associations between adherence to the Mediterranean diet and the results of assisted reproduction techniques (*n* = 8)

Authors, year	Country	Age range	Sample size (*n*)	Study type	Determination of adherence to the MD	Variables analysed	Results	Conclusions	Quality of Evidence (GRADE)^a^
Noli *et al.* (2023) [25]	Italy	18–39 years	296 women	Cross-sectional	Adherence to MD was assessed using the Mediterranean diet adherence score (MDS) [26] developed by Trichopoulou *et al.* in 2003 modified to adapt it to Italian dietary habits.	Poor ovarian response (retrieval of three or fewer suitable oocytes) in patients without criteria/risk factors for it.	The risk of poor ovarian response was significantly lower for women in the second tertile of MDS compared to the first tertile (OR = 0.29, CI_95%_ = 0.11–0.76) and for women in the second and third tertiles, grouped together, compared to the first tertile (OR = 0.34, CI_95%_ = 0.14–0.82).	Low adherence to the MD could be a risk factor for poor ovarian response in patients without criteria/risk factors for it.	Low ⊕⊕⊝⊝
Vujkovic *et al.* (2010) [18]	Netherlands	23–45 years	161 women	Cohort (prospective)	A 195-item FFQ with 22 food groups was used to assess dietary intake during the last 4 weeks. Subsequently, a principal component analysis was applied to the food groups for the construction of dietary patterns.	1) Fertilization rate 2) Embryo quality 3) Biochemical pregnancy	High adherence to the MD increased the likelihood of pregnancy (OR = 1.4, CI_95%_ = 1.0–1.9).	Following the MD prior to conception by women undergoing IVF/ICSI treatment is associated with success in achieving pregnancy.	Low ⊕⊕⊝⊝
Karayiannis *et al.* (2018) [19]	Greece	22–41 years	244 women	Cohort (prospective)	A questionnaire was used to assess adherence to the MD (PMD) [27] previously developed by Panagiotakos *et al.*, in 2007.	1) Ovarian stimulation results 2) Fertilization rate 3) Embryo quality 4) Clinical pregnancy 5) Live birth	Low adherence to MD was associated with a lower probability of achieving a clinical pregnancy (RR = 0.35, CI_95%_ = 0.16–0.78, *P* = 0.013) and live births (RR = 0.32, CI_95%_ = 0.14–0.71, *P* = 0.007).	Higher adherence to the MD is associated with a greater likelihood of clinical pregnancy and live births after IVF/ICSI treatment in non-obese women <35 years of age.	Low ⊕⊕⊝⊝
Gaskins *et al.* (2019) [28]	USA	18–46 years	357 women	Cohort (prospective)	A 131-item FFQ was used to assess dietary intake over the past year. Subsequently, MDS [26] was calculated to evaluate adherence to the MD.	1) Fertilization rate 2) Number of mature oocytes 3) Implantation rate 4) Clinical pregnancy 5) Live birth	Women in the higher quartiles (second to fourth) of MD adherence had a higher probability of live births (0.44, CI_95%_ = 0.39–0.49) compared to women in the lowest quartile (first quartile; 0.31, CI_95%_ = 0.25–0.39). No additional benefit was observed beyond the second quartile (*P* > 0.05).	Adherence to the MD, although associated with a higher probability of live births in the upper quartiles, may not be the most appropriate dietary guideline for all women undergoing fertility treatment in USA.	Low ⊕⊕⊝⊝
Ricci *et al.* (2019) [29]	Italy	27–45 years	474 women	Cohort (prospective)	A valid and reproducible 78-item FFQ was used that assessed the diet followed during the past year. Adherence to MD was assessed using Alternative Mediterranean diet index (AMD) developed by Fung *et al.* in 2009 [30].	1) Number of retrieved high-quality oocytes 2) Embryo quality 3) Clinical pregnancy 4) Live birth	No significant association was found between the MD and ART outcomes. The only exception was observed in women >35 years with intermediate adherence to MD, who showed a lower risk of not achieving a clinical pregnancy (RR = 0.84, CI_95%_ = 0.71–1.00, *P* = 0.049).	No clear association was observed between adherence to the MD and successful IVF.	Very low⊕⊝⊝⊝
Sun *et al.* (2019) [31]	China	18–40 years	590 women	Cohort (prospective)	A 69-item FFQ was used, and then adherence to the MD was evaluated using the MDS [26].	1) Embryo quality 2) Implantation rate 3) Number of fertilized oocytes 4) Clinical pregnancy	The group with higher adherence to the MD showed a greater number of available embryos (*P* = 0.028). In additional correlation tests, the number of fertilized oocytes (r = 0.089, *P* = 0.039) and embryo production (r = 0.102, *P* = 0.018) correlated positively with adherence to the MD.	Infertile women with higher adherence to the MD were likely to obtain more available embryos in the IVF cycle.	Low ⊕⊕⊝⊝
Salas-Huetos *et al.* (2022) [32]	USA	18–45 years	245 men	Cohort (prospective)	Dietary intake of men was assessed using a validated 131-item FFQ that analysed intake during the past year. Subsequently, three scores were calculated based on the FFQ: (1) MDS [26], (2) PMD [27], (3) AMD [30].	1) Fertilization rate 2) Implantation rate 3) Clinical pregnancy 4) Live birth	There was a marginally significant inverse association between adherence of men to the PMD score and fertilization rates (*P* < 0.05). Nevertheless, there were no significant associations of men’s adherence to any of the analysed dietary patterns with the probabilities of implantation, clinical pregnancy, or live birth.	Men’s adherence to the MD is not significantly related to major ART outcomes such as implantation, clinical pregnancy, or live birth.	Low ⊕⊕⊝⊝
Salas-Huetos *et al.* (2023) [33]	USA	18–45 years	612 women	Cohort (prospective)	The pre-treatment diet of the women was assessed using a validated 131-item FFQ that analysed dietary intake over the past year. Subsequently, three indices were calculated based on the FFQ: (1) MDS [26], (2) PMD [27], (3) AMD [30].	1) Clinical pregnancy 2) Total pregnancy loss 3) Clinical pregnancy loss 4) Live birth	There was no association between women's adherence to MD and the probability of clinical pregnancy or live birth after IVF or AI (*P* > 0.05).	The findings of this study suggest that there is no association between adherence to the MD and ART outcomes.	Low ⊕⊕⊝⊝

a GRADE = Grading of Recommendations, Assessment, Development, and Evaluation; ⊕⊕⊕⊕: high quality, randomized controlled trials with few limitations and strong associations. Future research is unlikely to change our confidence in the effect estimate; ⊕⊕⊕⊝: moderate quality, randomized controlled trials with some inconsistencies and/or a present dose-response gradient. Future research is likely to change our confidence in the effect estimate; ⊕⊕⊝⊝: low quality, randomized controlled trials with very important uncertainty and/or multiple confounding factors, and observational studies. It is very likely that future research will have an impact on our confidence in the effect estimate; ⊕⊝⊝⊝: very low quality, observational studies with multiple biases. Any estimate of the effect is very uncertain.

MD = Mediterranean diet; MDS = Mediterranean diet score; OR = odds ratio; CI_95%_ = 95% confidence interval; FFQ = food frequency questionnaire; IVF = *in vitro* fertilization; ICSI = intracytoplasmic sperm injection; PMD = Panagiotakos Mediterranean diet index; RR = relative risk; AMD = alternative Mediterranean diet index; ART = assisted reproduction techniques; r = Pearson correlation coefficient; AI = artificial insemination.

### Determination of adherence to the MD

Table 1 summarizes how studies assessed MD adherence, the variables analysed for ART outcomes, key findings, and conclusions. All studies used a food frequency questionnaire (FFQ) and applied an established index to evaluate MD adherence. Most (62.5%) [25, 28, 31–33] used the Mediterranean diet score (MDS) by Trichopoulou *et al.* (2003) [26] which evaluates nine components: vegetables, legumes, fruits, cereals, fish, monounsaturated fats, meat, dairy, and alcohol. One study (12.5%) [25] adapted the MDS for Italian dietary habits.

Three studies (37.5%) [19, 32, 33] used the Panagiotakos Mediterranean diet score (PMD), which assesses adherence to the MD through eleven components: cereals, potatoes, vegetables, legumes, fruits, nuts, fish, meats, dairy, alcohol, and fats [27]. Similarly, 37.5% of the studies [29, 32, 33] employed the alternative Mediterranean diet index (AMD) by Fung *et al.* (2009) [30], tailored for non-Mediterranean populations. Lastly, Vujkovic *et al.* [18] utilized an FFQ with 195 items across 22 food groups, adjusting for energy intake and applying principal component analysis to identify two dietary patterns: MD and ‘health-conscious, minimally processed’.

### Variables analysed to determine ART outcomes

In terms of the variables analysed to determine ART outcomes, there was some heterogeneity across studies. Some studies evaluated intermediate outcomes (number of mature oocytes and fertilization rate), and others assessed clinical endpoints (implantation, clinical pregnancy, and live births), or both. Among the studies that assessed intermediate outcomes of ART, four out of the eight analysed articles (50.0%) used embryo quality as a variable (validated morphokinetic parameters of embryonic development) [18, 19, 29, 31], four of them (50.0%) evaluated the fertilization rate [18, 19, 28, 32], and three studies (37.5%) examined the number of mature oocytes [28, 29, 31]. Additionally, two out of the eight included studies (25.0%) [19, 25] took into account the results of ovarian stimulation, and one study (12.5%) [18] considered biochemical pregnancy (a very early miscarriage confirmed by pregnancy hormone levels) as an outcome of ART.

Regarding clinical assessment criteria, six out of the eight analysed studies (75.0%) evaluated clinical pregnancy as an outcome [19, 29, 31–33], three articles (37.5%) [28, 31, 32] analysed the implantation rate, and five of them (62.5%) used live birth as a variable [19, 28, 29, 32, 33]. Finally, the study by Salas-Huetos *et al.* (2023) [33] also utilized total pregnancy loss, defined as a human chorionic gonadotropin (β-hCG) serum level exceeding 6 mIU/ml without confirmation of the presence of an intrauterine gestational sac at 6 weeks of gestation, and clinical pregnancy loss as variables to measure the outcome of ART.

### Relationship between adherence to the MD and ART outcomes

In Table 1, the results and conclusions of the studies included in this review are also presented. In conclusion, four out of the eight analysed articles (50.0%) [18, 19, 25, 31] suggested that associations exist between the degree of adherence to MD and ART outcomes. Conversely, four of the included articles (50.0%) found no statistically significant association [28, 29, 32, 33].

Specifically, among the articles that found associations between the degree of adherence to the MD and ART outcomes, the study by Vujkovic *et al.* (2010) [18] concluded that high adherence to the MD increased the likelihood of biochemical pregnancy (a very early miscarriage confirmed by pregnancy hormone levels) [odds ratio (OR): 1.5] after IVF/ICSI. On the other hand, Karayiannis *et al.* (2018) [19] observed that non-obese women under 35 years old with low adherence to the MD had a 65% lower probability of achieving clinical pregnancy (*P* = 0.01) and live births (*P* = 0.01). However, this study found no significant associations between the MD and intermediate study outcomes, such as ovarian stimulation results, fertilization rate, and measures of embryo quality. Finally, the study by Noli *et al.* (2023) [25] found that low adherence to the MD could be a risk factor for poor ovarian response in patients without criteria for it, a crucial aspect for successful IVF.

On the other hand, the study by Gaskins *et al.* (2019) [28] found no association between adherence to the MD and ART outcomes. However, this study investigated various dietary patterns and concluded that higher adherence to the ‘fertility-promoting’ diet was linearly associated with a significantly higher likelihood of implantation, clinical pregnancy, and live births (trend *P* < 0.001 for all). This diet had a slight similarity to MD, as the study described it as a dietary pattern rich in folic acid, vitamin B12, vitamin D, agricultural products low in pesticide residues, whole grains, dairy, soy, and seafood. In turn, Ricci *et al.* (2019) [29] and Salas-Huetos *et al.* (2023) [33] also found no association between adherence to the MD pattern in infertile women and ART outcomes. Finally, in the study by Salas-Huetos *et al.* (2022) [32], in which they analysed the effect of adherence to various dietary patterns, including the MD (determined through various previously validated indices), they found an inverse association between men’s adherence to the MD and the fertilization rate. However, there were no statistically significant associations between men’s adherence to any of the analysed dietary patterns and the odds of implantation, clinical pregnancy, or live birth.

### Risk of bias assessment

**Figure 3.**
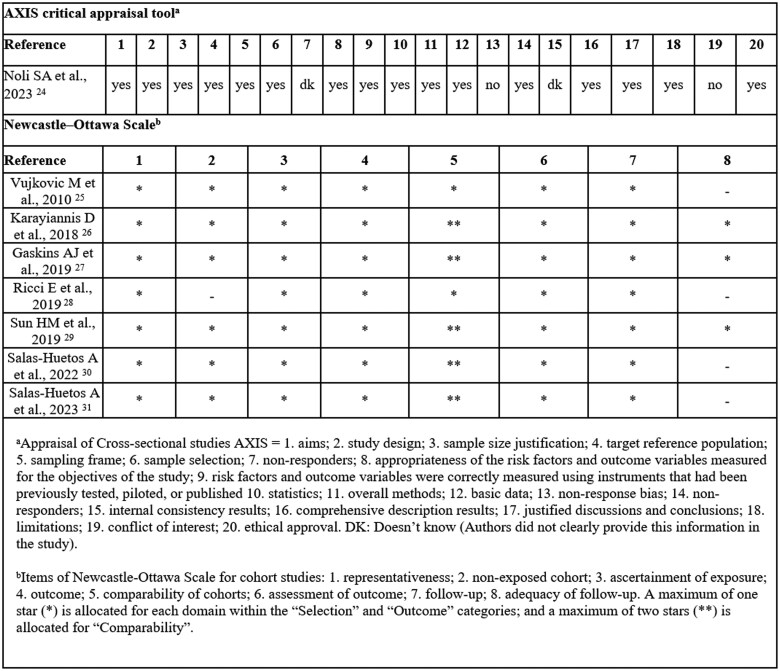
Critical appraisal of studies included in the systematic review of associations between Mediterranean diet adherence and assisted reproductive technology outcomes using the AXIS tool and the Newcastle-Ottawa scale.

Figure 3 illustrates the risk of bias in cross-sectional studies assessed with the AXIS scale [22]. The study by Noli *et al.* (2023) [25] showed moderate risk due to unspecified details and unaddressed nonresponse bias and conflicts of interest. Figure 3 also includes the NOS [23] for cohort studies, as no case-control studies were reviewed. Overall, cohort studies had a low risk of bias, though four out of seven studies [18, 29, 32, 33] showed bias in sample follow-up adequacy.

### Quality of the evidence

In Table 1, the quality of evidence of the included articles was also analysed based on the GRADE model, which is highly relevant in evidence-based medicine [24]. In this regard, seven out of the eight included studies (87.5%) [18, 19, 25, 28, 31–33] had low-quality evidence, and one of them (12.5%) [29] had very low-quality evidence. As can be seen, all studies had low or very low quality because they were observational studies with the presence of some biases.

## Discussion

### Main findings

The objective of this systematic review was to evaluate the associations of adherence to MD in infertile couples on ART outcomes. Among the eight articles included in this review, four of them reported statistically significant associations between adherence to the MD and outcomes (both intermediate and clinical) of ART. Specifically, positive associations were found between higher adherence to the MD pattern and the likelihood of having a greater number of embryos available in the IVF cycle [31], biochemical pregnancy [18] (a very early miscarriage confirmed by pregnancy hormone levels), clinical pregnancy [19], and live births [19]. Similarly, low adherence to the MD was statistically linked to a poor ovarian response during the IVF cycle in patients without criteria for it [25].

On the other hand, regarding studies that did not find significant associations between ART outcomes and the MD, several relevant aspects are noteworthy. First, one study [28] found that greater adherence to a dietary pattern with a slight similarity to that of MD, the ‘pro-fertility’ diet, was associated with a higher probability of live births after ART. Secondly, in the study by Ricci *et al.* (2019) [29], the findings showed that adherence to the MD was not significantly associated with IVF outcomes. However, an exception was observed in women over 35 years with moderate adherence to the MD, who had a lower risk of not achieving clinical pregnancy. Additionally, the only study exclusively investigating the influence of MD adherence in a sample of men also found no association with relevant ART outcomes [32].

### What is already known on this subject?

After conducting the bibliographic search, it was observed that research in this area was limited, mostly focusing on isolated nutrients or foods, and presenting inherent limitations in accurately measuring dietary patterns [20]. Most of the studies were cohorts, and there was some heterogeneity in both the instruments used to measure adherence to MD and the variables used to determine ART outcomes [20]. Similarly, as observed in this review, studies evaluating the influence of the MD on ART outcomes primarily focused on women’s adherence to this dietary pattern, neglecting the potential impact of men’s or the complete couple’s diet.

On the other hand, after an exhaustive review of the scientific literature, there were studies evaluating the effect of the MD on various parameters of reproductive health, showing positive results in terms of infertility prevention or endocrine disorders linked to it. For example, a case-control study from the University of Navarra’s Follow-up Project (SUN) [34], analysing a sample of 485 infertile women and 1669 controls, determined that high adherence to the MD was associated with a higher likelihood of becoming pregnant. Other studies assessing semen quality found that adherence to the MD was positively associated with sperm concentration and total count [35], as well as sperm motility [16]. An inversely proportional association was also found between adherence to the MD pattern and the clinical severity of polycystic ovary syndrome, the most common endocrine disorder linked to female infertility [36, 37]. Similarly, a case-control study conducted in Italy concluded that high vegetable consumption, adherence to the MD, and a low dietary inflammatory index (DII) were related to a lower risk of endometrial cancer [38, 39]. This study also emphasized the importance of nutritional status, as excess body fat contributes to hormonal dysregulation and increased endometrial inflammation, increasing the risk of this type of cancer [38]. Overall, most research highlighted that the positive effects of the MD seemed to be mediated by its ability to improve nutritional status and reduce oxidative stress (OS)—a state characterized by an imbalance between pro-oxidant and antioxidant molecules—chronic low-grade inflammation, and insulin resistance, conditions linked to endocrine/metabolic disorders and infertility in both sexes [38–40].

In this context, several authors have highlighted that inflammation is an increasingly recognized factor affecting reproductive health and contributing to what is known as ‘inflammatory infertility’ [40]. A study conducted in 2023, evaluating the DII in a sample of 4437 participants, concluded that having a proinflammatory diet increased the likelihood of infertility in women by 86%. Likewise, postprandial hyperglycaemia, caused by the consumption of large amounts of high-glycaemic-index carbohydrates, has been shown to be associated with increased inflammation and OS. Additionally, insulin has a direct influence on ovarian function, and hyperinsulinemia is closely related to hyperandrogenism, aggravating endocrine disorders in women, and making conception more difficult. Similarly, OS, characterized by an imbalance between prooxidative and antioxidative molecules, plays a crucial role in the pathogenesis of infertility in both men and women. In part, this explains why the MD, characterized by its abundance of anti-inflammatory nutrients (omega-3 fatty acids, vitamin C, selenium, polyphenols…) antioxidants, and low-glycaemic-index carbohydrates, has been shown to offer benefits in reproductive health that can be extrapolated to the success of ART. However, as reflected in this review, evidence regarding its potential association on ART outcomes is still scarce to definitively confirm its clinical benefits in this specific area.

### Strengths and limitations of the review

This review has several limitations. Only full-text articles published in English or Spanish were included, which may have led to the exclusion of relevant studies in other languages. The search strategy, though systematic and structured using MeSH terms, may have missed some evidence not indexed with those terms. Additionally, variability in the tools used to measure MD adherence and ART outcomes introduces heterogeneity, and the inclusion of only observational studies limits the capacity to establish causality.

Nonetheless, this review also has important strengths. It is the first to synthesize current evidence on the association between adherence to the MD and ART outcomes using a systematic approach. A rigorous study selection process was conducted, and the methodological quality of the included studies was evaluated using validated tools. Furthermore, the focus on the MD as a whole dietary pattern enables a more comprehensive and realistic interpretation of the findings, compared to reviews centered on isolated nutrients or supplements. This approach aligns with the growing interest in dietary patterns and their role in reproductive health. Finally, by compiling findings from different populations and study designs, this review contributes to a broader understanding and may inform future dietary interventions in fertility care.

## Conclusions

Evidence suggests a possible link between adherence to the MD and ART outcomes, though causality cannot be established due to the observational nature of the studies. Limited research in this area highlights the need for further studies, particularly on the MD’s anti-inflammatory effects, its role in infertility mechanisms, and its influence on assisted reproduction outcomes.

## Supplementary Material

ckaf100_Supplementary_Data

## Data Availability

This is a systematic review of previously published studies. No new data were generated or analysed in this study. All data used are available in the original publications included in the review. Key points Adherence to the MD shows potential positive associations with ART outcomes, including a higher number of embryos, biochemical pregnancy, clinical pregnancy, and live births. Limited evidence and the observational nature of existing studies prevent establishing causality, highlighting the need for further research on the MD’s role in infertility mechanisms and ART outcomes. Inconsistent findings across studies may stem from variations in the tools used to assess adherence to the MD and differences in study populations.
