# Supplementary material for: Associations between adherence to a Mediterranean diet and assisted reproductive techniques outcomes: a systematic review
Source: Eur J Public Health. 2025 Jul 11;35(5):960–9. doi: 10.1093/eurpub/ckaf100 (PMC12529289; doi:10.1093/eurpub/ckaf100)
Supplement: ckaf100_Supplementary_Data [file ckaf100_supplementary_data.docx]

**Supplementary References**

The following references (41–47) are provided as supplementary material, in accordance with the journal’s reference limit policy.

1. Ricceri F, Giraudo MT, Fasanelli F, et al. Diet and endometrial cancer: a focus on the role of fruit and vegetable intake, Mediterranean diet and dietary inflammatory index in the endometrial cancer risk. *BMC Cancer.* 2017;17:757. <https://doi.org/10.1186/s12885-017-3754-y>
2. Cirillo M, Argento FR, Becatti M, et al. Mediterranean Diet and Oxidative Stress: A Rela-tionship with Pain Perception in Endometriosis. *Int J Mol Sci.* 2023;24(19):14601. <https://doi.org/10.3390/ijms241914601>
3. Alesi S, Villani A, Mantzioris E, et al. Anti-Inflammatory Diets in Fertility: An Evidence Review. *Nutrients.* 2022;14(19):3914. <https://doi.org/10.3390/nu14193914>
4. Moludi J, Kamari N, Darbandi M, et al. Association between dietary inflammatory index and infertility of women; Results from RaNCD Cohort Study. *Nutr J.* 2023;22:35. <https://doi.org/10.1186/s12937-023-00865-6>
5. Łakoma K, Kukharuk O, Śliż D. The Influence of Metabolic Factors and Diet on Fertility. *Nutrients.* 2023;15(5):1180. <https://doi.org/10.3390/nu15051180>
6. Mínguez-Alarcón L, Chavarro JE, Gaskins AJ. Caffeine, alcohol, smoking, and reproductive outcomes among couples undergoing assisted reproductive technology treatments. *Fertil Steril.* 2018;110(4):587-592. <https://doi.org/10.1016/j.fertnstert.2018.05.026>
7. Arhin SK, Zhao Y, Lu X, et al. Effect of micronutrient supplementation on IVF outcomes: a systematic review of the literature. *Reprod Biomed Online.* 2017;35(6):715-722. <https://doi.org/10.1016/j.rbmo.2017.08.018>
